# Climate change and health modeling: horses for courses

**DOI:** 10.3402/gha.v7.24154

**Published:** 2014-05-23

**Authors:** Kristie L. Ebi, Joacim Rocklöv

**Affiliations:** Epidemiology and Global Health, Department of Public Health and Clinical Medicine, Umeå Centre for Global Health Research, Umeå University, Umeå, Sweden

**Keywords:** climate change, climate variability, modeling, health impacts, research needs

## Abstract

Mathematical and statistical models are needed to understand the extent to which weather, climate variability, and climate change are affecting current and may affect future health burdens in the context of other risk factors and a range of possible development pathways, and the temporal and spatial patterns of any changes. Such understanding is needed to guide the design and the implementation of adaptation and mitigation measures. Because each model projection captures only a narrow range of possible futures, and because models serve different purposes, multiple models are needed for each health outcome (‘horses for courses’). Multiple modeling results can be used to bracket the ranges of when, where, and with what intensity negative health consequences could arise. This commentary explores some climate change and health modeling issues, particularly modeling exposure-response relationships, developing early warning systems, projecting health risks over coming decades, and modeling to inform decision-making. Research needs are also suggested.

The current and projected human health consequences of climate change are diverse and wide-ranging, potentially altering the burden of any health outcome sensitive to weather or climate. Climate variability and change can affect morbidity and mortality from extreme weather and climate events, and from changes in air quality arising from changing concentrations of ozone, particulate matter, or aeroallergens (1). Altering weather patterns and sea level rise also may facilitate changes in the geographic range, seasonality, and incidence of selected infectious diseases in some regions, such as malaria moving into highland areas in parts of sub-Saharan Africa (1–3). Changes in water availability and agricultural productivity could affect undernutrition, particularly in parts of Asia and Africa (4). The pathways between climate change and these health outcomes are often complex and indirect.

Excluding the direct health burdens from extreme weather and climate events, the standard for evidence-based exposure-response relationships for climate-sensitive health outcomes differs from that for many environmental and occupational exposures. No health care use or death certificate will have climate change as an underlying or contributing cause; instead, the records focus on the physiological mechanism(s) associated with the event. Further, these health outcomes generally have multiple and often interacting drivers of their geographic range, incidence, and seasonality; factors that are infrequently recorded on patient care records and death certificates. These records and death certificates are not designed to consider that climate change may have contributed to a case of (or death from) diarrheal disease by increasing the rate of pathogen replication in warmer ambient temperatures or by facilitating the distribution of pathogens by increased heavy precipitation events.

Given the many and complex linkages between climate processes and a health outcome, understanding the extent to which weather, climate variability, and climate change are affecting and may affect future health burdens in the context of other risk factors, and the temporal and spatial patterns of any changes, is challenging if not impossible without developing mathematical and statistical models. Ideally, such models are based on the association between the exposure and outcome, taking into consideration the relevant processes and drivers, and possible sources of confounding and bias. The question is not whether to model, but whether there are sufficient data over long-enough time periods and robust enough understanding of the processes relating the climate factors to health outcomes to develop useful models and, if so, how to model. If appropriately designed and communicated effectively, policy- and decision-makers can use health models integrating micro- to macro-level exposures and processes that influence the occurrence of a health outcome to select a basket of policies and measures to efficiently and effectively manage health risks over shorter and longer temporal scales.

Scientific curiosity alone does not drive modeling the health risks of current and projected climate change. Models are designed to answer specific policy-relevant questions. Two principal aims are to guide adaptation measures to increase resilience, and to guide mitigation policies to reduce greenhouse gas emissions, thereby reducing health risks past mid-century. Common purposes for health models are to understand exposure-response relationships (based on biological or statistical relationships), estimate the relative contributions of different risk factors, map the locations of populations vulnerable to particular health outcomes or environmental conditions, develop early warning systems, and project longer-term health risks under different climate and socioeconomic scenarios. The data needs, the spatial and temporal scales, and the model structure and robustness vary across models, with no one model able or robust enough to answer all questions at all scales.

*Horses for courses* is an idiom that captures that multiple models are needed for each health outcome of concern because different models serve different uses, with each model better than other models at addressing a particular issue. Models developed for one purpose may not be appropriate for other purposes. In fact, it is highly unlikely that a model developed as the basis of an early warning system, which thus contains sufficient detail and contextual information to be able to forecast where and when health risks could increase under particular environmental conditions in a specific location, would also be appropriate for projecting how health risks could evolve over this century under different climate and socioeconomic scenarios at a range of spatial and temporal scales.

Considerations in model development include quantification of the climate–health associations and the factors that affect those associations; specification of model(s) appropriate to incorporate climate variability and change, adaptation, and mitigation policies, including consideration of temporal and spatial scale issues; incorporation of thresholds; incorporation of pathways of public health development; and quantification of uncertainties (5). The description of a model should include the conceptual framework underlying its structure, key assumptions, and its temporal and spatial scale. The usefulness of a model and its output of current or future climate-sensitive health burdens depend on not only the robustness of the model, but also the extent to which the model provides results that address the needs of stakeholders, at the appropriate scales of interest.

This commentary explores some issues with modeling the health risks of climate change, particularly modeling exposure-response relationships, developing early warning systems, projecting health risks over coming decades, and modeling to inform decision-making. We conclude with some suggested research needs.

## Modeling exposure-response relationships

Public health and health-care organizations demand, appropriately so, quantitative evidence of the associations between risk factors and health outcomes, and of the effectiveness, including costs and benefits, of interventions before modifying current or implementing new measures (e.g. early warning systems) to reduce exposures and vulnerabilities. Long-term data sets are required to estimate the contributions of various factors to health burdens. Even when such data sets are available, the complexities of interactions in the chain of causation for a climate-sensitive health outcome mean that modeling is often the best approach for understanding how and to what extent weather and climate patterns contribute to health outcomes, in the context of multiple drivers, and for furthering understanding of possible interventions to reduce health risks. For example, increasing summertime ambient temperatures increases the risks of heat-related morbidity and mortality. The urban surrounding, including built infrastructure (e.g. green roofs, location of cooling centers), the presence of trees, and other factors can modify hot temperatures, making some locations within a community warmer and others cooler. The extent to which individuals are sensitive to hot temperatures depends on their level of fitness, presence of certain chronic diseases, use of drugs that affect thermal regulation, clothing choices, and other factors. Understanding the relative importance of different factors through modeling can be helpful for targeting interventions to reduce morbidity and mortality.

In biological and process-based models, clearly described causal interrelationships are used to simulate disease dynamics. Statistical models often have different requirements. Data-driven models can be developed in cases where the underlying disease processes are not well understood or well quantified, and where there is, for example, insufficient information to develop a causal pathway model. In such cases, a simple model would always be preferable to a more complex model given similar predictive ability. It is always important for models to be validated using data not used in model fitting to avoid over-fitting and to increase confidence in the predictive accuracy in new situations.

## Mapping vulnerable regions and using exposure-response relationships to develop early warning systems

Mapping can be very helpful for local and national decision-makers to enhance current health protection, and can be used to support vector and disease surveillance and early warning systems on the timing and scale of vector control operations and public health interventions, more effectively deploying scarce human and financial resources. Early warning systems are based on forecasts of how the numbers of cases vary by levels of environmental variables, balancing the need for the time needed to prepare for an outbreak with the skill of the forecast over different temporal scales.

For example, the increasing worldwide burden of dengue fever has led to interest in mapping where dengue is currently (or may soon be) transmitted. The distribution of dengue depends not just on the temperature and precipitation requirements and constraints of the vector and pathogen, but also on vector control and surveillance programs (and their effectiveness), awareness and effectiveness of health-care organizations in diagnosis and treatment, how travel and trade affect vector and pathogen distribution, and urbanization patterns. Dengue early warning systems are important for managing outbreaks, particularly with the lack of a vaccine or treatment options. For example, weekly mean temperature and cumulative rainfall in combination with disease surveillance can be used to forecast dengue epidemics 16 weeks in advance in Singapore (6).

One of the challenges with the models underlying early warning systems is that they assume that historic distribution patterns and relationships will remain constant in the future. However, this may not always be the case. For example, increasing ambient temperatures will lead to acclimatization that could alter the relationships between heat waves and morbidity and mortality. For infectious diseases, changing disease transmission dynamics and control policies means that models are likely to need adjustment to maintain forecast precision as weather patterns shift over time.

## Applying models in other regions or at other scales

The use of a model needs to be consistent with its development. For example, a model validated in one community over a particular time period to support an early warning system may not provide valid predictions in other communities or at other temporal scales. Although an early warning system can provide guidance on key factors and approaches for developing such a system elsewhere, analyses will be needed of how local transmission patterns, key contextual factors, and other variables could alter the early warnings or responses.

There is often a trade-off between increasing the goodness of fit of a model versus improving its predictive ability. For example, risk maps of malaria and dengue using spatial information of reported disease frequency are sometimes validated locally by matching to a nearby nontransmission zone, including matching on local weather patterns. Such models are trained to distinguish local differences in the predictor variables on the disease outcome but are not trained to discriminate global influences that may alter disease risks, such as longer-term changing weather patterns with climate change (7). Therefore, such models should not be used for global projections of risk or incidence under climate change. Also, a model that effectively discriminates on local features may be ineffective or misleading if applied at the global level. Similarly, model validation to one location provides no guarantee that using the model for another location would be appropriate because contextual factors can indirectly influence model parameters. For global (local) predictions and projections, models validated with global (local) data are the most accurate.

## Projecting possible future health risks

While increasingly detailed understanding of the determinants of a climate-sensitive health outcome on short time scales can enhance the effectiveness of public health interventions, this level of detail may not be needed or useful to provide realistic projections of disease burdens in mid-century and beyond under different possible futures. Projecting the extent to which alterations in weather patterns may affect future health burdens requires moving beyond models only based on exposure-response relationships and projected temperature/precipitation change to models that incorporate a range of plausible (and relevant) environmental and socioeconomic futures. For example, variables that can influence the burden and pattern of vector-borne diseases include weather patterns, land use, demographic growth, and factors associated with socioeconomic development, such as the status of the health system, the availability of human and financial resources, diagnostic and treatment technologies, and others.

Models can be used to identify which of the many complex interrelationships and interdependencies that determine a health outcome are the most important to describe its current geographic range, seasonality, and incidence, and to project how these might change when one or more parameters change. Not all drivers of a climate-sensitive health outcome are of equal importance for understanding the role(s) of climate variability and change. Models can provide insights into which variables and interactions are critical.

Lyme disease is an example of a disease where increasing model complexity is not always needed. Lyme disease has a multi-year life cycle that includes four life stages: egg, six-legged larva, eight-legged nymph, and adult. Blood meals are required during the last three stages for tick survival and reproduction. Human pathogens acquired during feeding can be transmitted at the next feeding stage. Ogden et al. (8) reviewed knowledge of the drivers for changes in the geographic range of ticks and tick-borne pathogens via effects on their basic reproduction number *R*_0_, and the mechanisms of dispersal that allow ticks and tick-borne pathogens to invade suitable environments. *R*_0_ identifies when the Lyme pathogen, *Borelia burgdoferi*, can spread when introduced into a fully susceptible population. Therefore, *R*_0_ is an index of species fitness in current or possible future locations. Many observed patterns are consistent with expectations from theoretical studies or studies of other species. Key factors determining the expansion and invasion of *Ixodes scapularis* ticks and *B. burgdoferi* are changes in climate, habitat, and host. Dunn et al. (9) developed a mechanistic model of whether *B. burgdoferi* can spread when introduced into a fully susceptible population. Nineteen parameters of biological relevance were included, taking into account the transmission efficiency from the vertebrate host as a function of the days since infection. Three factors were the most influential in terms of their contribution to the observed variation in *R*_0_: transmission efficiency from the vertebrate hosts to *I. scapularis* ticks (which depends on the innate susceptibility of the host community to infection), tick survival rate from fed larva to feeding nymph (largely determined by ambient temperature), and the fraction of nymphs finding a competent host (largely determined by the composition of the host community and the relative densities of reservoir host species). Given the widespread distribution of possible hosts, modeling how climate change could alter the geographic range of Lyme disease can therefore be simplified to focus on temperature, as the susceptibility and composition of the host community may not be constraints when the vector and pathogen expand into new regions.

## Modeling to inform policy- and decision-making

Insights gained from modeling how climate change could affect future health burdens at local to regional scales, and across near-term to longer time slices, are important for prioritizing adaptation and mitigation strategies to increase the resilience of future societies, particularly for processes with longer-term planning horizons. For example, sea level rise, storm surges, and increasingly heavy precipitation may mean that hospitals need to be moved from flood-prone areas. Models can indicate the timing, location, and level of possible risks, and likely safe distances from coastlines, major rivers, and other sources of flooding risk. Modeling also can quantify the extent to which urban design could increase active transport possibilities, reduce flooding risks, and result in other co-benefits.

Model development should consider not only what scientists know about weather/climate–health relationships, but also what stakeholders need to know for effective decision-making (10). The purpose of projecting health burdens is to gain insights into general trends and patterns, including key interactions and dynamics, under different scenarios of future climate and development pathways. Doing so provides decision-makers with a broad-brush vision of how health burdens could change to inform longer-term planning. Because the future is unknown, one should view projections not as precise estimates of what will happen, but as more order-of-magnitude estimates of what could happen if the underlying assumptions of climate and development are met.

Policymakers can use model results to identify mitigation and adaptation targets, and approaches to meeting those targets that promote human health and well-being. To be most effective, modeling should be an iterative process involving scientists and policymakers to ensure that results contribute to the body of scientific knowledge and address the needs of decision-makers. National and international assessments often facilitate this upstream/downstream communication by identifying not only key findings from published literature, but also knowledge gaps that, if filled, would enhance the policy relevance of research.

Because each model projection only captures a narrow range of possible futures, a necessary investment for managing the health risks of weather, climate variability, and climate change is to have multiple models of when, where, and with what intensity negative health consequences could arise over shorter and longer time scales, to bracket the ranges of future health burdens to which regions and nations will need to adapt and mitigate. Any one model may miss the key dynamics affecting future uncertainties. Similar motivations have led to the development of multiple models in other sectors as well as model intercomparison projects (e.g. the World Climate Research Program for earth system models (http://www.wcrp-climate.org/) or the Agricultural Model Intercomparison and Improvement Project (AgMIP; www.agmip.org) that includes activities for model intercomparisons and improvement).

## Research needs

Developing conceptual frameworks and modeling of the interrelationship among the determinants of a health outcome can identify research needs by highlighting where additional information could significantly improve understanding that can then, in turn, improve model validity and usefulness. Models, from exposure-response relationships to projections of the health risks of climate change, are needed to explore the range of potential impacts of weather and climate in the context of other drivers of population health. Modeling should be a core activity in research centers and public health organizations to facilitate understanding and risk management of the health risks of climate change.

While considerable progress has been made in understanding the risks of extreme ambient temperatures and how changing weather patterns could affect the geographic range, seasonality, and incidence of some infectious diseases, much more needs to be learned to develop exposure-response relationships to underpin effective public health interventions, including early warning and response systems. Low funding levels means efforts are limited for developing models that explore how drivers of climate-sensitive health outcomes interact under a range of possible future climatic and socioeconomic conditions to further planning for managing future health risks. Models are needed to explore not just how climate change could impact individual health outcomes, but also possible impacts across interacting health outcomes, such as diarrheal disease, malaria, and undernutrition.

Developing modeling protocols and guidelines, including the types of models appropriate for particular purposes, considerations of geographic and temporal scales, approaches to validation, and handling of uncertainties, would enhance the usefulness and relevance of models. Doing so would increase comparability, facilitating intermodel comparisons. Malaria is one of the few health outcomes modeled by more than one research group, which provided a recent opportunity for an intermodel comparison (2). This paper clearly shows that major contributors to the variability in the projected risks include conceptual differences in the models, model parameterization, whether the model was developed for early warning or to project risks in a changing climate, and local to global validation schemes. Developing more consistent approaches to modeling malaria risk would increase confidence in the robustness of results found by multiple research groups, and likely decrease the inconsistencies across global impact projections that at least partially arise from using models developed for different purposes. Further, models projecting risks should better incorporate nonclimatic drivers of future vulnerability, for example, based on the Shared Socioeconomic Pathways (11).

Model results should be communicated across local-to-national scales, to build capacity of relevant stakeholders at all levels, including research, public health and health-care managers, user communities, and the public. Collaboration across these communities is needed to iterate to effective solutions to manage current disease burdens from climate change, taking the local context into consideration, and to continue iterating as further climate change requires adjustments to models and public health and health-care programs to prepare for and cope with projected risks.
